# Polarization-Controlled and Flexible Single-/Penta-Band Metamaterial Absorber

**DOI:** 10.3390/ma11091619

**Published:** 2018-09-05

**Authors:** Jiayun Wang, Rongcao Yang, Jianping Xu, Jinping Tian, Runbo Ma, Wenmei Zhang

**Affiliations:** School of Physics and Electronic Engineering, Shanxi University, Taiyuan 030006, China; w_jiayun@163.com (J.W.); xjpsxu@163.com (J.X.); tianjp@sxu.edu.cn (J.T.); marunbo@sxu.edu.cn (R.M.); zhangwm@sxu.edu.cn (W.Z.)

**Keywords:** metamaterial absorber, polarization-controlled, penta-band, flexibility

## Abstract

In this paper, a polarization-controlled and flexible metamaterial absorber made of a set of wires etched on ultrathin teflon dielectric substrate is proposed. The simulation results showed that the proposed absorber achieved single-band absorptivity of 99.8% at 6.64 GHz for the TM (transverse magnetic) polarization wave and penta-band absorptivity of more than 99% at 11.68 GHz, 13.58 GHz, 15.48 GHz, 17.38 GHz, and 19.28 GHz for the TE (transverse electric) polarization waves. Moreover, each absorption peak had very narrow relative bandwidth and the position of penta-band absorption peaks could be adjusted by changing the length of the corresponding wire or selecting suitable substrate material according to actual requirements, because each wire can independently respond to electromagnetic (EM) waves. Furthermore, the surface current distributions corresponding to each absorption peak were studied to demonstrate the absorption mechanism. The absorption properties of the proposed structure with different bending radii and under different incident angles of the EM waves were investigated, showing good flexibility and incident angle-insensitive properties. In addition, the simulation results were confirmed by measuring a fabricated prototype. The proposed absorber may have useful applications in polarizers, sensors, bolometers, polarization detectors, etc.

## 1. Introduction

Metamaterials (MMs) are a kind of artificial composite material consisting of periodic unit cells smaller than the wavelength of incident electromagnetic (EM) waves. The EM parameters of MMs can be artificially engineered by designing the shape and size of the unit structure, resulting in fascinating physical characteristics and phenomena such as a negative refractive index, inverse Doppler effect, invisibility cloaks, and perfect absorption [1,2,3,4,5]. MM absorbers are generally designed to match the impedance of free space by tailoring the effective permittivity and permeability, thus the EM wave can entirely enter the absorber, and the EM power can be dissipated by dielectric losses and ohmic loss [5]. Since Landy et al. first realized a perfect microwave absorber made of split ring resonators in theory and experiment [5], the MM absorbers have garnered great interest due to the potential applications in imaging, sensors, solar cells, and radomes, etc. Compared to conventional absorbing materials such as wedge foam, ferrite, graphite, Salisbury screens, and others, MM absorbers have many notable merits including being thin, light weight, low cost, and having high absorptivity [6,7,8,9,10].

Polarization characteristics are important in the design of the absorber, as they are elementary properties of EM waves. Most researchers are devoted to designing polarization-independent MM absorbers using rotational symmetry structures such as nested square rings [11,12], circumscribed-cross resonators [13], hexagonal unit cells [14,15], cross-cave patches [16], circular sectors [17,18], rotational petal structure [19], and bow-tie array [20]. Recently, several switchable polarization-insensitive absorbers were designed by loading symmetric diodes and a biasing network [21,22]. However, in order to manipulate the absorbers to respond to different polarized EM waves, the polarization-controlled MM absorbers have been proposed [23,24,25,26,27]. For instance, Hokmabadi et al. proposed a single-band polarization-dependent, stereometamaterial, and perfect absorber composed of two tightly coupled nonconcentric rings [23]. Tuong et al. and Kim et al., respectively, realized dual-band polarization-controlled absorbers with double concentric rings structure [24] and four unsymmetrical cut wires [25]. Wang et al. proposed a triple-band polarization-tunable absorber composed of a patch structure with two indentations spaced by an insulating layer [26]. Tang et al. achieved a polarization-controlled broadband absorber by vertically placing nine different size resistive films on a metal plate [27]. Most recently, in order to meet the specified requirements such as a curved surface or irregular surface, some works focused on the flexibility of MM absorbers with good absorption performance by adopting some dielectric substrates with excellent flexibility, such as polydimethylsiloxane (PDMS) [20,28], polyimide [29,30], teflon [31], felt [32], ultrathin FR-4 [33], and silicone rubber [34]. It should be noted that most of the aforementioned flexible/nonflexible, polarization-controlled/independent absorbers have fewer absorption bands, which greatly restricts their potential applications. Although some triple or four-band absorbers were proposed by nested structures or horizontal/vertical arrangement of resonant cells with different sizes [11,35,36,37], with similar structural design ideas, it is difficult to propose an absorber with smaller unit size and more absorption bands. The motivation of this paper is to design a novel flexible absorber with simple structure, small unit size, polarization-controlled multiple absorption bands, and easily adjustable absorption frequencies by engineering the geometrical parameters or selecting suitable materials for different situations.

In this paper, we designed a simple MM absorber composed of a set of wires etched on one side of ultrathin teflon dielectric substrate and a copper plate covered on the other side of the substrate. Such an MM absorber can switch between single-band and penta-band perfect absorption through EM polarization. The proposed absorber exhibited a single-band absorption with absorptivity of 99.8% for the TM (transverse magnetic) polarized wave at 6.64 GHz and penta-band absorption with absorptivity of more than 99% for the TE (transverse electric) polarized wave at 11.68 GHz, 13.58 GHz, 15.48 GHz, 17.38 GHz, and 19.28 GHz. Also, the absorber possesses good flexibility with good absorption property when bent to a certain extent due to the ultrathin thickness of 1/48 and 1/140 of the minimum and maximum operating wavelength. Moreover, the proposed absorber shows wide incidence angle insensitivity to both TE and TM polarized waves. In order to explore the absorption mechanism of the proposed MM absorber, the surface current distributions at the frequency of each absorption peak were analyzed. Finally, the prototype of the absorber was prepared by circuit board etching technique and measured for different polarizations and curvatures in free space. The measured results are in good agreement with those of the simulation.

## 2. Structure, Simulated, and Experimental Setup

**Figure 1:**
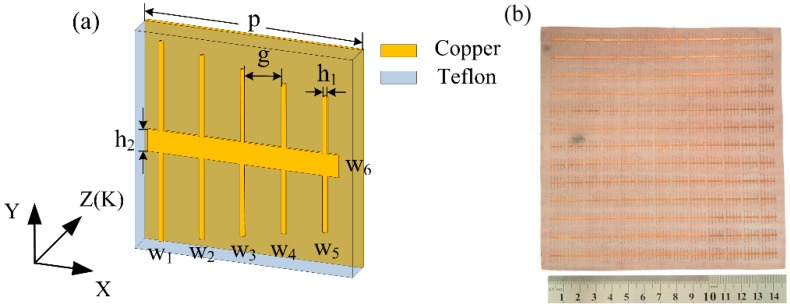
(**a**) Unit cell schematic diagram of the proposed absorber; (**b**) Photograph of fabricated prototype.

The unit cell of the proposed absorber is shown in Figure 1a; a teflon dielectric substrate was adopted. The absorber has the following measurements: Young modulus *E* = 0.5 KN/mm^2^, thickness *d* = 0.25 mm, relative permittivity *ε* = 2.1, tangent loss tan*δ* = 0.0002, and the periodic dimension in X-Y plane *p* = 11 mm. The front side of the dielectric substrate is etched with a copper resonator pattern that consists of five vertical wires (w_1_–w_5_) and one horizontal wire (w_6_), the back side is covered by a copper plate whose thickness and conductivity are *h* = 0.036 mm and *σ* = 5.8 × 10^7^ s/m, respectively. In addition, the other optimized dimensions of the unit cell are as follows: the lengths and widths of the wires are, respectively, *l*_1_ = 9.16 mm, *l*_2_ = 8.4 mm, *l*_3_ = 7.6 mm, *l*_4_ = 6.8 mm, *l*_5_ = 6.16 mm, *l*_6_ = 9.8 mm, *h*_1_ = 0.2 mm, *h*_2_ = 1.0 mm, and the distance between adjacent wires is *g* = 1.9 mm.

The performance of the proposed absorber in Figure 1a was studied by numerical simulation and experimental measurement. The simulation was performed through the EM software CST 2015 (CST China, LTD., Shanghai, China) based on the standard finite integration technology (FIT), we set unit cell boundary conditions in the X and Y directions, and an open (add space) boundary for the Z direction. The normally incident EM wave was along the Z axis, which was vertical to the absorber surface, and the electric and magnetic fields were polarized at the Y and X directions, respectively. The absorptivity *A*(*ω*) can be calculated from the extracted reflection coefficient *S*_11_ and the transmission coefficient *S*_21_ as follow:

(1) $$A(\omega )=1-R(\omega )-T(\omega )=1-{\left|S_{11}\right|}^{2}-{\left|S_{21}\right|}^{2},$$

Since the back side of the substrate is coated with copper, the incident waves are completely blocked. Therefore, the perfect absorptivity can be achieved when the reflectivity is close to zero. The reflectivity *R*(*ω*) is given by:

(2) $$R(\omega )=\frac{Z(\omega )-Z_{0}}{Z(\omega )+Z_{0}},$$

where *Z*(*ω*) and *Z*_0_ are the impedances of the absorber and the free space, respectively. *Z*(*ω*) can be adjusted by tailoring the relative permittivity *ε_r_*(*ω*) and permeability *μ_r_*(*ω*) as follow [2]:

(3) $$Z(\omega )=\sqrt{\frac{\mu_{0}\mu_{r}(\omega )}{\varepsilon_{0}\varepsilon_{r}(\omega )}},$$

where *ε*_0_ and *μ*_0_ are the permittivity and the permeability of free space, respectively. So through elaborative design, when *ε_r_* is sufficiently close to *μ_r_* at the resonant frequencies, the impedance match condition *Z*(*ω*) ≈ *Z*_0_ will be achieved. In this case, the incident EM wave will completely enter the interior of the absorber where the EM energy can be dissipated by the ohmic losses and dielectric losses. Therefore, the absorber will perfectly absorb EM waves without reflection and transmission.

In the experiment, we fabricated a 143 mm^2^× 143 mm^2^ prototype with 13 × 13 resonant units by means of a circuit board etching process, where the geometrical parameters and the material parameters of the fabricated prototype are the same as those in the simulation. The non-bended photograph of the prototype is shown in Figure 1b. As a copper plate on the back is used to block the transmission of EM waves, we just needed to measure the reflectivity. We adopted the NRL (Naval Research Laboratory) arch reflectivity testing method [38,39] to measure the reflectivity of the prototype in free space. The vector networker analyzer (Agilent N5230A, Agilent Technologies, Santa Clara, CA, USA) was connected to the transmitting and receiving antennas which were mirrored on both sides of the incident normal line. The prototype was located at the center of the arch and surrounded by wedge foam absorbing materials. When measuring the prototype under normal incident, the angle between the antennas and the incident normal was set to about 2° to avoid the influence of the antennas. Here, the distance between the antennas and the prototype was set to satisfy the far-field condition. In order to measure the polarization characteristics, the prototype was rotated by 0°, 30°, 60°, and 90° for different polarization angles. As the adopted teflon dielectric substrate was very thin, the absorber prototype could be bent to a certain extent. The prototype was bent with different curvature radii to measure bent situations. Before each measurement, we calibrated the experimental instrument with the copper plate being the same size as the absorber prototype. Thus, the actual results were obtained through comparing the measured reflection coefficient of the prototype with that of the copper plate.

## 3. Results and Discussion

**Figure 2:**
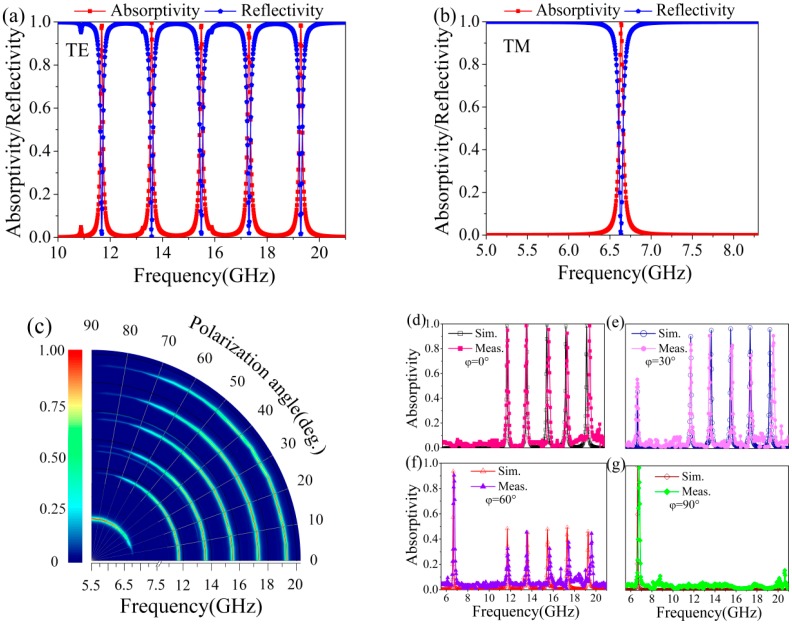
The absorptivity under normal incident for (**a**) TE mode; (**b**) TM mode; (**c**) simulation, and (**d**–**g**) contrast of simulated and measured results in non-bended situation for different polarization angles.

Figure 2 shows the absorption performance of the proposed absorber under normal incident. It can be seen from Figure 2a that for the TE polarization wave, the absorptivity measurements were 99.25%, 99.81%, 99.13%, 99.01%, and 99.55% at the frequencies 11.68 GHz, 13.58 GHz, 15.48 GHz, 17.38 GHz, and 19.28 GHz with the relative absorption bandwidth 0.87%, 0.82%, 0.79%, 0.73%, and 0.66%, respectively. However, for the TM polarization wave, these five absorption peaks were replaced by one peak at 6.64 GHz with the absorptivity of 99.8% and the relative bandwidth of 0.81%, as shown in Figure 2b. Figure 2c illustrates the simulated absorptivity under different polarization angles *φ*, which is defined as the angle between the electric field E and the X axis direction. It is easy to see from Figure 2c that the absorptivity of five resonance frequencies is gradually reduced with the increase of polarization angle and is switched off when the polarization angle *φ* is 90°. Meanwhile, the absorptivity of the single absorption peak at 6.64 GHz is gradually increased, and it will achieve nearly 100% when *φ* is 90°. Figure 2d–g depict the contrast curves of the measurement and simulation results in the non-bended situation under different polarization angles 0°, 30°, 60°, and 90°, respectively. They clearly show that the simulation and measurement results are in good agreement with each other except for the slight deviation coming from manufacturing accuracy and testing error. This implies that the absorption peaks of the proposed absorber can be controlled by the polarization angle.

**Figure 3:**
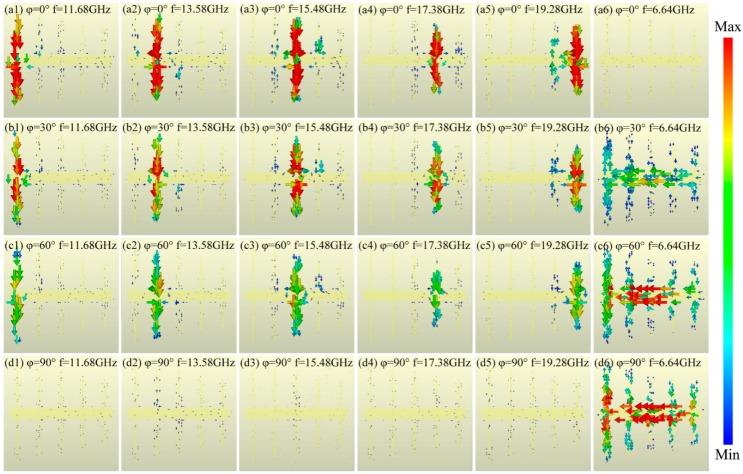
The surface current distributions at the absorption peaks under different polarization angles *φ* of (**a1**–**a6**) 0°, (**b1**–**b6**) 30°, (**c1**–**c6**) 60°, and (**d1**–**d6**) 90°.

To understand the physical absorption mechanism of the proposed absorber, the surface current distributions at each resonance frequency are demonstrated in Figure 3. As shown in Figure 3a1–a6, for polarization angle 0°, i.e., electric field direction along the X axis, the five vertical wires independently respond to the EM wave, generating five pairs of anti-parallel currents between the wires and the copper plane at 11.68 GHz, 13.58 GHz, 15.48 GHz, 17.38 GHz, and 19.28 GHz, thus five independent absorption peaks are formed at corresponding response frequencies. When the polarization angle *φ* increases to 30°and 60°, at corresponding response frequencies, the currents on the vertical wires become weaker, but grow stronger on the horizontal wire, as shown in Figure 3b1–b6 and c1–c6, respectively. This is because the electric field component decreases gradually along the X axis, while increasing gradually along the Y axis. When the polarization angle increases to 90°, i.e., electric field direction along the Y axis, the horizontal wire strongly responds to the EM wave at a single absorption peak at 6.64 GHz, while little response occurs on the five vertical wires at the other frequencies, as shown in Figure 3d1–d6. Thus, such a proposed structure exhibits the polarization-controlled single-/penta-band absorption property.

The performance of the proposed absorber was compared with that of the previous polarization-controlled absorbers, as listed in Table 1. It is clear that the proposed absorber operates in different frequency ranges from the previous structures that have at most three polarization-controlled absorption peaks, while the proposed structure can realize a switch between one and five absorption peaks by polarization control. Moreover, its absorptivity of all peaks is above 99%, which is comparable to or slightly better than the previous publications.

Furthermore, it is noted from Figure 3 that the different length of wires w*_i_* (*i* = 1, 2, …, 6) can respond to different frequency bands, because the response frequency is proportional to the distance through which the equivalent current flows [40]:

(4) $$\omega \propto c/\pi l_{i}\sqrt{\varepsilon_{r}}$$

where *c* is the velocity of light in a vacuum and *l_i_* = (*i* = 1, 2, …, 6) is the length of the wires.

**Figure 4:**
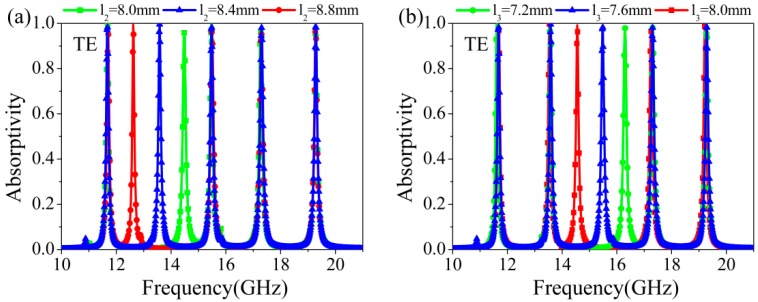

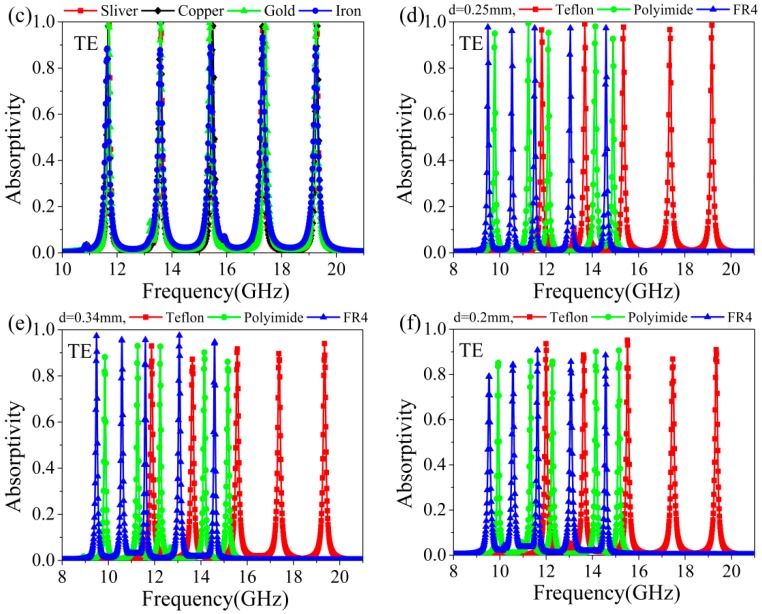
The absorptivity for different wire lengths (**a**) *l*_2_ and (**b**) *l*_3_, (**c**) for different metals, (**d**–**f**) for different dielectric materials with different thickness.

In this regard, we investigated the influence of the wire length on absorptivity by taking w_2_ and w_3_ as examples, as shown in Figure 4a,b. It can be clearly seen that the corresponding red absorption peak or blue shifts while the other absorption peaks are almost unaffected when the length *l*_2_ or *l*_3_ increases or decreases. This means that the coupling between these wires is very little, and each wire of the proposed structure can independently absorb the EM wave at the corresponding frequency. In other words, the position of absorption peaks can be accordingly adjusted by simply changing the length of the wires according to actual requirements. Also, we explored the effect of different metallic and dielectric materials on the performance of the proposed MM absorber. Figure 4c shows the absorptivity for the different adopted metallic materials of wires and the covered plate as silver, copper, gold, and iron with conductivities of 6.3 × 10^7^ s/m, 5.8 × 10^7^ s/m, 4.56 × 10^7^ s/m, 1.04 × 10^7^ s/m, respectively. It can be observed that the metallic material slightly influences the position of the absorption peaks, while the materials with a smaller conductivity may lead to a decrease of the absorptivity. Figure 4d–f show the absorptivity for the different dielectric materials teflon, polyimide, and FR-4 with the permittivity measurements of 2.1, 3.5, and 4.4, and different thicknesses of 0.25 mm, 0.3 mm, and 0.2 mm, respectively. It can be seen from Figure 4d that for the optimized thickness *d* = 0.25 mm, the proposed structure adopting teflon, polyimide, and FR-4 dielectric substrates can have the absorptivity of above 96%, but larger permittivity leads to a red shift of the absorption peaks, just as Formula (4) suggests. When we increased or decreased the thickness of dielectric substrate, *d* = 0.34 mm and *d* = 0.2 mm, respectively, the absorptivity of the absorber decreased to different extents, as shown in Figure 4e,f. This is because the thickness of the absorber depends the geometry of the adopted resonant unit cells [10], which implies that in order to achieve high absorptivity, the thickness of the absorber must match the shape and size of the resonant unit cells. Therefore, one can engineer the position of the absorption peaks of the designed MM absorbers by engineering the geometrical parameters and selecting suitable materials for different situations.

**Figure 5:**
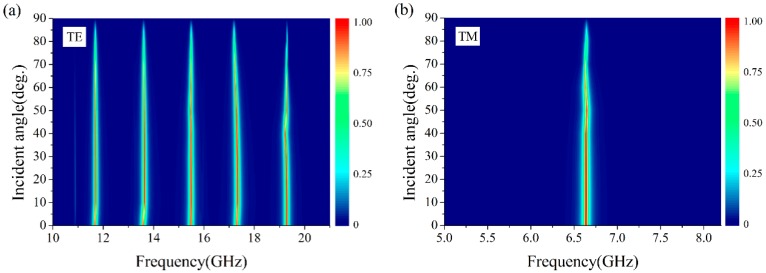
The absorptivity for (**a**) TE polarization mode and (**b**) TM polarization mode with different incident angle *θ*.

In practical application, the EM waves are not always normal incident to the absorber surface, so the absorptivity for wide angle incidence is an important aspect for evaluating the performance of an absorber. We simulated the absorptivity of the proposed absorber for TE and TM polarization waves with different incident angle *θ*, as shown in Figure 5. It is clearly observed that the absorptivity of the proposed absorber is retained at over 90% for both TE and TM polarization waves when the incident angle is increased to 60°.

**Figure 6:**
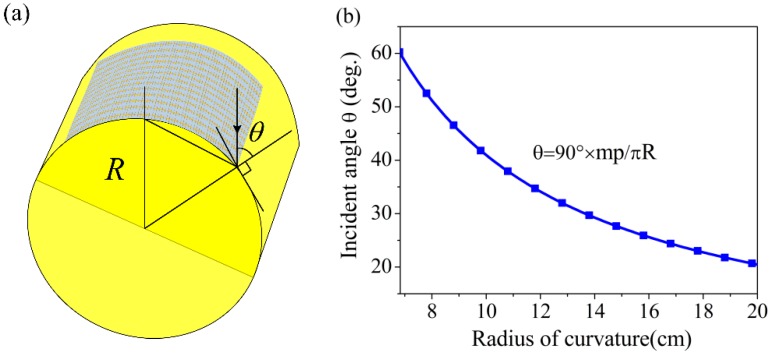
(**a**) Schematic diagram of the proposed absorber attached on the cylinder with radius of curvature R, (**b**) the relationship curve between R and the incident angle *θ* with m = 13.

In addition, due to the flexibility of the teflon dielectric substrate and the thickness of the proposed absorber being almost 1/48 and 1/140 of the minimum and maximum operating wavelength, the absorber can be bent to conform to some curved surfaces. Therefore, we investigated the absorption property by bending the proposed structure as a cylindrical surface, as illustrated in Figure 6a. Effectively, for a normal incident EM wave, the radius of the curvature R and the incident angle have a relation *θ* = 90° × *mp/πR*, where m is the unit cell number of the prototype. Figure 6b depicts the relationship of the curvature radius R and the incident angle *θ*. Combining the angle-insensitive absorption property shown in Figure 5, it is easy to see that when the curvature radius *R* ≥ 3*mp*/2*π* corresponding to *θ* ≤ 60°, the bent proposed absorber will still have good absorptivity for both TE and TM waves.

**Figure 7:**
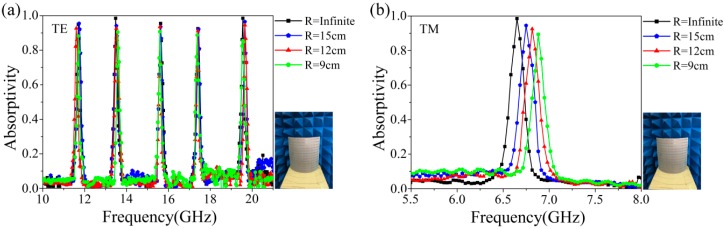

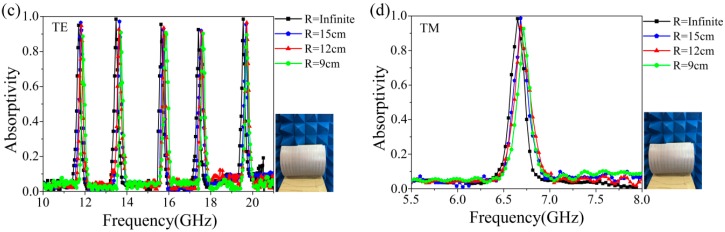
The measured absorptivity under different bent situations. (**a**,**b**) along the X axis; (**c**,**d**) along the Y axis.

For the fabricated prototype, it is easily to see from Figure 6b that when the radius of curvature is greater than 6.83 cm, the bent absorber has good absorption performance. Furthermore, we measured the absorptivity of the bent prototypes with curvature radii of 15 cm, 12 cm, and 9 cm as shown in Figure 7. For comparison, the absorption curve of the unbent prototype with R = infinite is also presented. Because of the asymmetry of the structure, we measured the absorptivity under the different bent direction along the X and Y axis. For the bent direction along X axis, the measured results for both TE and TM polarization wave are illustrated in Figure 7a,b, when the radius of curvature is decreased to R = 9 cm, the all absorption peaks are maintained above 84% for both TE and TM polarization waves. The bent case has little influence on response frequency of the five absorption peaks, while the single absorption peak has a blue shift with the decrease of curvature radius. This is because the equivalent length of the w_6_ is reduced when the absorber is bent along X axis, and the response frequency increases according to formula (4). However, when the bent direction is in the Y axis, the absorption performances are less affected except for a little blue shift, and the absorptivity of all peaks is about 90%, as shown in Figure 7c,d. These measured results show that the proposed absorber can retain high absorptivity when it is bent to a certain extent.

## 4. Conclusions

In conclusion, we propose a polarization-controlled and flexible single-/penta-band MM absorber composed of a set of wires. The results show that the absorber can exhibit multi-band absorptivity of 99.25%, 99.81%, 99.13%, 99.01%, and 99.55% at 11.68 GHz, 13.58 GHz, 15.48 GHz, 17.38 GHz, and 19.28 GHz, respectively, for TE waves, and single-band absorptivity of 99.8% at 6.64 GHz for TM waves. Also, the single-band and multi-band absorption can be controlled by the EM polarization, and all the relative absorption bandwidths are very small. The position of penta-band absorption peaks can be engineered by changing the length of the wires or selecting suitable substrate materials according to actual requirements, because each wires can independently respond to EM waves. In order to explain the absorption mechanism, the corresponding surface current distributions at each absorption peak under different polarization modes are presented. Meanwhile, we demonstrated good angular insensitivity and flexibility of the proposed absorber by investigating absorption performance under different incident angles of the EM wave and under different bending radii of the proposed structure along X and Y axis directions. In addition, we fabricated a prototype with 13 × 13 resonant units and confirmed the simulated results by the experimental measurement. The absorber is thin and has a simple design, and it is polarization-controlled, flexibile, and has very small relative absorption bandwidths. The proposed absorber is expected to have useful applications in polarizers, sensors, bolometers, polarization detectors, etc.

## Figures and Tables

**Table 1:** Performance comparisons of the proposed absorber with previous works.

Reference	Frequency	Polarization-Controlled Peaks	Absorptivity
23	0.75–0.81 THz	1 to 1	98.1–98.7%
24	8.55–11.78 GHz	2 to 1	96.0–98.0%
25	6.46–10.89 GHz	2 to 2	99.91–99.99%
26	0.96–2.95 THz	3 to 3	97.7–99.8%
27	21.0–52.2 GHz	1 to 1	90.0–99.91%
Proposed design	6.64–17.38 GHz	5 to 1	99.01–99.81%
